# Operculectomy Preceding a Mucoepidermoid Carcinoma Development: Case Report

**DOI:** 10.1155/crid/1092442

**Published:** 2026-01-21

**Authors:** Torres Urbina Claudia Araceli, Liceaga Escalera Carlos Juan, Montoya Pérez Luis Alberto, Sosa Villanueva Brenda Vanessa, Aldape Barrios Beatríz Catalina

**Affiliations:** ^1^ Private Practice, Academic of UNAM, Oral Surgery Clinic, Universidad Nacional Autónoma de México, Mexico City, Mexico, unam.mx; ^2^ Oral and Maxillofacial Surgery Service, Hospital Juárez de México, Mexico City, Mexico, hospitaljuarez.salud.gob.mx; ^3^ Faculty of Oral and Maxillofacial Surgery Service, Hospital Juárez de México, Mexico City, Mexico, hospitaljuarez.salud.gob.mx; ^4^ Oral Surgery Clinic, Universidad Nacional Autónoma de México, Mexico City, Mexico, unam.mx; ^5^ Oral Pathology Academic, Universidad Nacional Autónoma de México (UNAM), Mexico City, Mexico, unam.mx

**Keywords:** mucoepidermoid carcinoma, oral cancer, oral surgery, pericoronitis

## Abstract

We present the case of an 18‐year‐old female patient with a history of multiple operculectomies that preceded the development of mucoepidermoid carcinoma in the retromolar region. Mucoepidermoid carcinoma is a malignant glandular epithelial neoplasm characterized by mucous, intermediate and epidermoid cells. Although its etiology is so far uncertain, there are various clinical manifestations that help us understand its biological behavior. The treatment depends on the TNM staging.

## 1. Introduction

Mucoepidermoid carcinoma (MEC) was first described in 1954 by Stewart, Foote, and Becker, who reported 45 of 700 tumors originating in the major and minor salivary glands. In this same year, Stewart introduced the term MEC to define a salivary gland neoplasm characterized by a mixed pattern made up of three main types of cells: epidermoid, intermediate, and mucus‐producing or mucosecretory cells [1].

## 2. Epidemiology

MEC accounts for less than 10% of all head and neck neoplasms, 10%–15% of all salivary gland neoplasms, and 30% of all salivary malignancies [2, 3].

MEC occurs more frequently in females, with prevalence between the third and fifth decades of life; being the most common malignant tumor in childhood–adolescence [1, 4], approximately 5% of these tumors occur in patients under 18 years of age [5].

### 2.1. Location

It´s found mainly in the parotid gland (56.8%); however, the hard palate is a frequent site, 18% when it occurs in the minor glands, intraorally in the retromolar trigone, floor of the mouth, oral mucosa, lip and tongue; and extraorally, it´s located in the submandibular and sublingual glands. The probability of malignancy in a parotid mass is 15%–32%; in a submandibular mass, it´s 41%–50%; in minor salivary gland masses, it´s 70%–90%; and close to 100% in sublingual masses [2, 6–9].

### 2.2. Etiology

Its origin has been described from the reserve cells of the excretory ducts, which are considered pluripotent cells [7]. It has been described that low‐dose radiotherapy multiplies the risk of development of neoplasia, use of radioactive iodine (thyroid pathology), entrapment of primordia of retromolar mucous glands, Epstein–Barr and human immunodeficiency viruses, immunosuppression, and exposure to ultraviolet light; unlike other head and neck carcinomas, tobacco and alcohol are not related to the development of the neoplasia [6].

In 2003, Tonon et al. associated MEC with genetic mutations that help its development; they speak of a t(11,19)(q21, p13) translocation, which fuses the CRTC1::MAML2 genes [10].

The fusion possibly disrupts the important NOTCH signaling pathway in intercellular communication. Present in 38%–81% in MEC, it has been seen that cases that have this translocation have a better prognosis since it´s associated with low‐grade neoplasms [2, 5, 10].

### 2.3. Clinical Features

Its clinical manifestations are diverse depending on the degree and location; they usually present as a rubbery or soft mass, painless, fixed to deep planes. Due to their superficial location, intraoral tumors may appear as enhanced blue–red staining that mimics a mucocele or vascular tumor. Occasionally, symptoms include ipsilateral ear discharge, dysphagia, lockjaw, and often facial paralysis [3, 6].

According to its biological behavior, it´s classified as high, intermediate, or low‐grade malignant neoplasm. The differences that we can find between a low‐grade one and a high‐grade one are that, in the low‐grade one, it´s growth is usually slow and painless; one could say that, clinically, it takes a course toward the benign [2], compared to that of the high grade that grows quickly, painfully, and with the presence of ulcerations [3].

Sublingual MEC can be painful, even when it´s small in size. Patients with sublingual tumors are generally detected quickly, due to existing symptoms in 80% of cases, and in parotid or submandibular tumors, there are only early symptoms in less than 33% of cases [3].

### 2.4. Histopathological Features

ESCs are solid, cystic, or microcystic growths that are frequently lobulated and circumscribed; their contours are invasive, irregular, stellate, or pointed.

Tumors in the retromolar area or hard palate may invade the underlying cancellous bone [2, 10].

Classical literature describes a subclassification according to its histopathological features into high, intermediate, and low grade. The predominant cells are mucosal, intermediate, and epidermoid. These cellular elements are arranged in nests and diffuse layers that can surround cystic spaces [2, 3, 10].

Mucous cells have a pyramidal, cup‐shaped, or spheroidal appearance; appear as heaped collections or individual units; and do not usually show atypia or mitotic activity. Intermediate cells are perceived in different ways; a variety of basaloid to columnar shapes have been considered. They often correspond to small, cubic cells with poorly defined edges. Squamous cells are polygonal and may present atypia or mitosis [2].

In low‐grade MEC, mucosal and intermediate cells with multiple cystic spaces predominate. On the other hand, high‐grade lesions are characterized by solid islets, fewer mucus‐secreting cells, and a high proportion of stratified squamous epithelial cells. Lesions of an intermediate grade are found between both extremes; mucous cells and cystic spaces are observed, but not as numerous as in high grade; they often have a larger population of intermediate cells [3, 5].

Low‐grade MEC arises most frequently in the minor salivary glands and is usually detected in earlier stages. High‐grade MEC, on the other hand, arises most frequently in the major salivary glands, especially the parotid gland [1].

### 2.5. Differential Diagnoses

Entities considered in low‐grade MEC include mucocele, necrotizing sialometaplasia, pleomorphic adenoma, sclerosing sialadenitis with mucosal metaplasia and Warthin′s tumor with oncocytic or squamous metaplasia, polycystic sclerosing adenoma, and secretory carcinoma. High‐grade MEC must be distinguished from carcinoma ex pleomorphic adenoma, poorly differentiated squamous cell carcinoma, adenosquamous carcinoma, salivary gland duct carcinoma, and metastatic carcinomas [3, 10].

### 2.6. Diagnosis

An incisional biopsy can be used for minor salivary glands in the oral cavity, but it´s not recommended for parotid lesions due to the risk of damage to the facial nerve and the possibility of tumor seeding. Therefore, ultrasound‐guided fine needle aspiration (FNA) is preferred [6].

### 2.7. Imagenology

Ultrasound is recommended for superficial lesions and major salivary gland lesions; it can help locate tumors, distinguish solid masses from chemical collections, and help guide the biopsy.

Computed tomography (CT) presents greater sensitivity in the evaluation of the involvement of bone structures.

Magnetic resonance imaging (MRI) is recommended to evaluate tumor extent, soft tissue invasion, and nerve involvement [6].

### 2.8. Treatment

For better management/treatment (Table 1), it´s divided into stages according to its extension, location, and degree of malignancy.

**Table 1:** Own resource.

**Staging of salivary gland cancers (8, 9)**		**Management strategy**
Stage I	Noninvasive tumors without spread to lymph nodes and without distant metastasis a surgical approach and/or radiotherapy (if high grade or if complete removal was not possible)	Surgical approach and/or radiotherapy (if high grade or if complete removal was not possible)
Stage II	An invasive tumor without spread to lymph nodes and without distant metastasis a surgical approach (may include removal of lymph nodes) and/or radiotherapy (if high grade)	Surgical approach (may include removal of lymph nodes) and/or radiotherapy (if high grade)
Stage III	Smaller tumors (< 4 cm) that have spread to regional lymph nodes, but without signs of metastasis. Extensive surgery (removal of the salivary gland where the carcinoma originates, nearby tissue and lymph nodes), which can be combined with radiotherapy or chemotherapy	Extensive surgery (removal of the salivary gland where the carcinoma originates, nearby tissue and lymph nodes) can be combined with radiotherapy or chemotherapy
Stage IV A	Any invasive tumor that does not involve lymph nodes or has spread to a single lymph node on the same side, but without metastasis. Extensive surgery, combined with radiotherapy, chemotherapy, and even clinical trial treatments	Extensive surgery, combined with radiotherapy, chemotherapy, and even clinical trial treatments
Stage IV B	Any cancer, with more extensive spread to lymph nodes, but without metastasis	
Stage IV C	Any cancer with distant metastases	

*Note:* Source: own resource using the data of the mentioned articles.

### 2.9. Prognosis

Low‐grade MECs have a good prognosis with a 5‐year survival rate greater than 90%, although recurrence is possible. A study carried out in 2014 with a sample of 2400 patients shows that intermediate and low‐grade tumors should be grouped together since there is no difference in survival.

The high‐grade variant of MEC has a high tendency for recurrence, cervical metastasis, and distant metastasis with a 5‐year survival as low as 30%–41.6% [2, 6].

## 3. Clinical Findings

**Figure 1:**
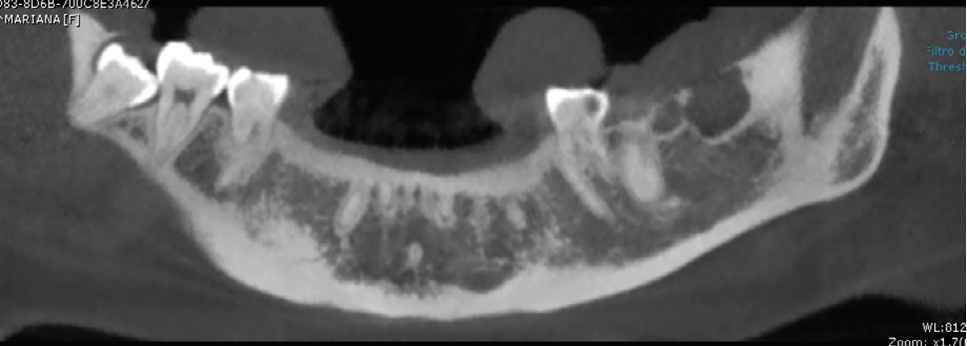
Cone beam with bone lysis is observed at the level of the retained third molar that extends toward the second molar and the posterior part of the mandibular body.

**Figure 2:**
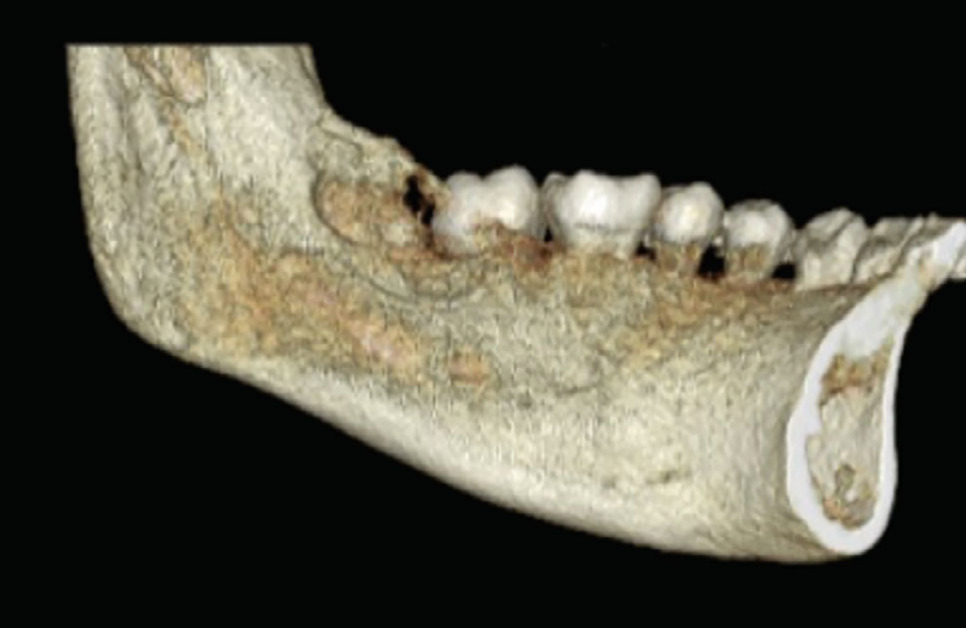
Three‐dimensional reconstruction with saucerization on the lingual surface of the mandibular body.

An 18‐year‐old female patient presents for consultation due to an increase in volume in the retromolar region of 1 year of evolution, with pain when chewing, with a positive surgical history of operculectomy on two occasions, and the patient reports that she was afraid of the wisdom tooth removal and she insists on only getting operculectomy. The relevant nonpathological history is that she has worked in a beauty salon since she was 15 years old; the rest of the history is not relevant to her current condition. Cone beam tomography was requested (Figures 1 and 2), where bone destruction was observed adjacent to the lower left third molar, with anterior extension to the second molar and toward the posterior part of the mandibular body.

An incisional biopsy is taken under local anesthesia (Figure 3); friable tissue with increased vascularity is observed; a surgical dressing is placed to cover the surgical bed and prevent bleeding, and it is sent for histopathological study where a specimen formed by multiple cavities is reported (Figure 4). Cystic cells with mucosecretory cells and islands of epithelioid cells with cellular and nuclear pleomorphism in a dense fibrous connective tissue with mild diffuse chronic inflammatory infiltrate and recent hemorrhage issued a diagnosis of low‐grade MEC. She was referred to the oncology center for treatment, where a posterior mandibular block resection with negative adjacent margins was performed, with follow‐up at the oncology center with no evidence of recurrence.

Figure 3(a) Exophytic, indurated mass with a sessile base, ulcerated, whitish, adjacent to the molar crown. (b) Biopsy taken for histopathological study, we observed a highly vascularized surgical bed. (c) Placement of surgical cement for hemostatic purposes.

**Figure:**
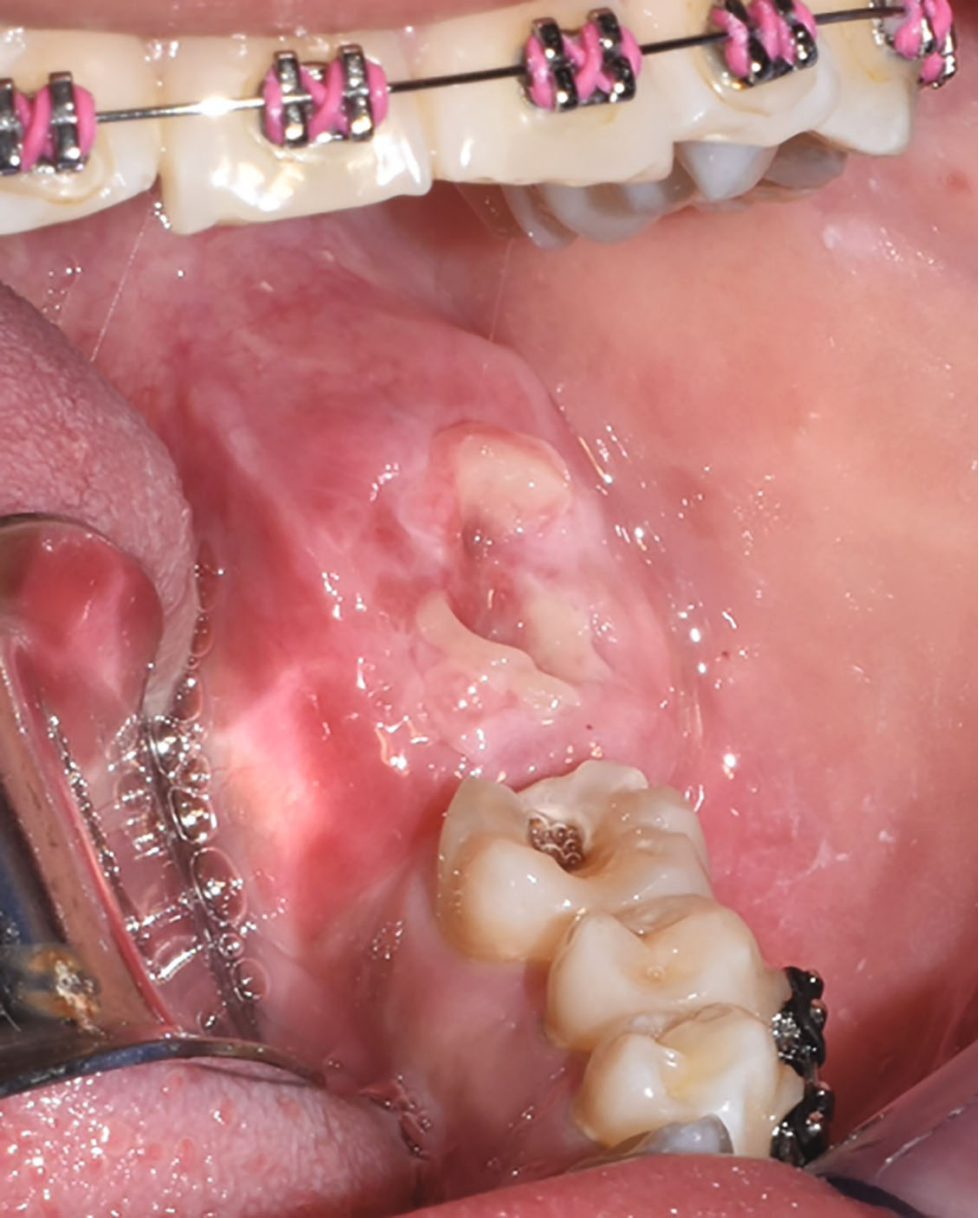
(a)

**Figure:**
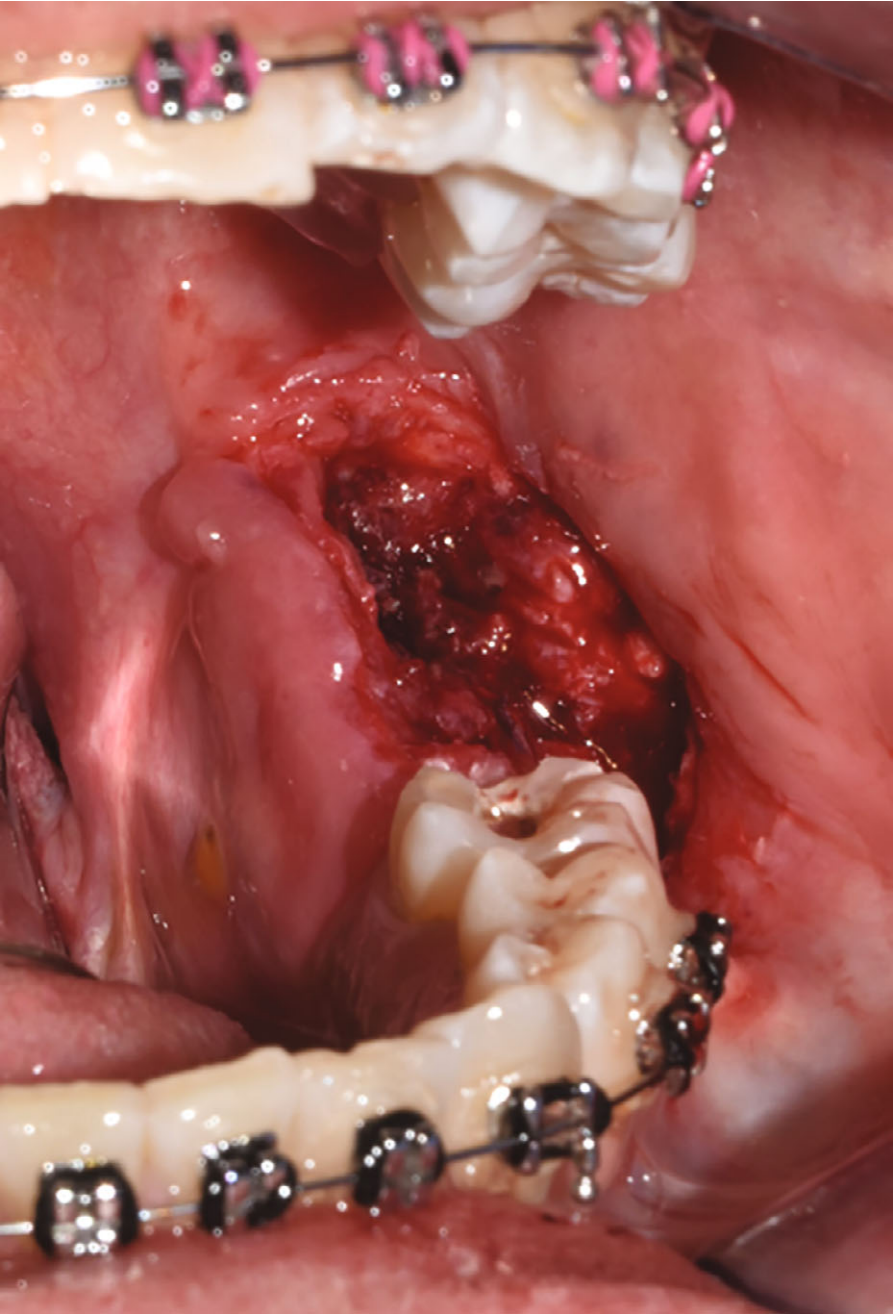
(b)

**Figure:**
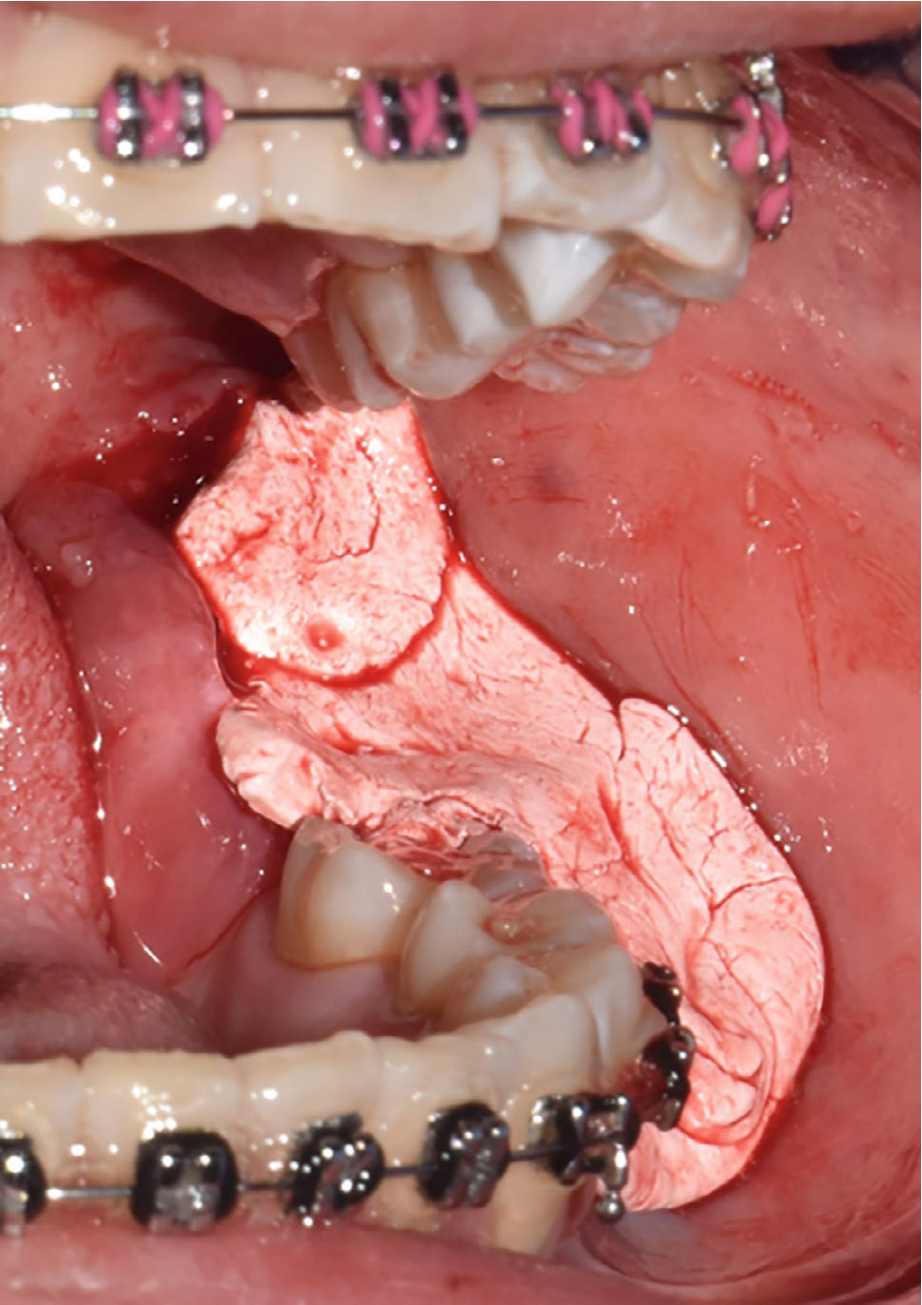
(c)

Figure 4(A) Histopathology 10× hematoxylin and eosin staining. (B) Section at 100×, where mucosecretory cells are observed.

**Figure:**
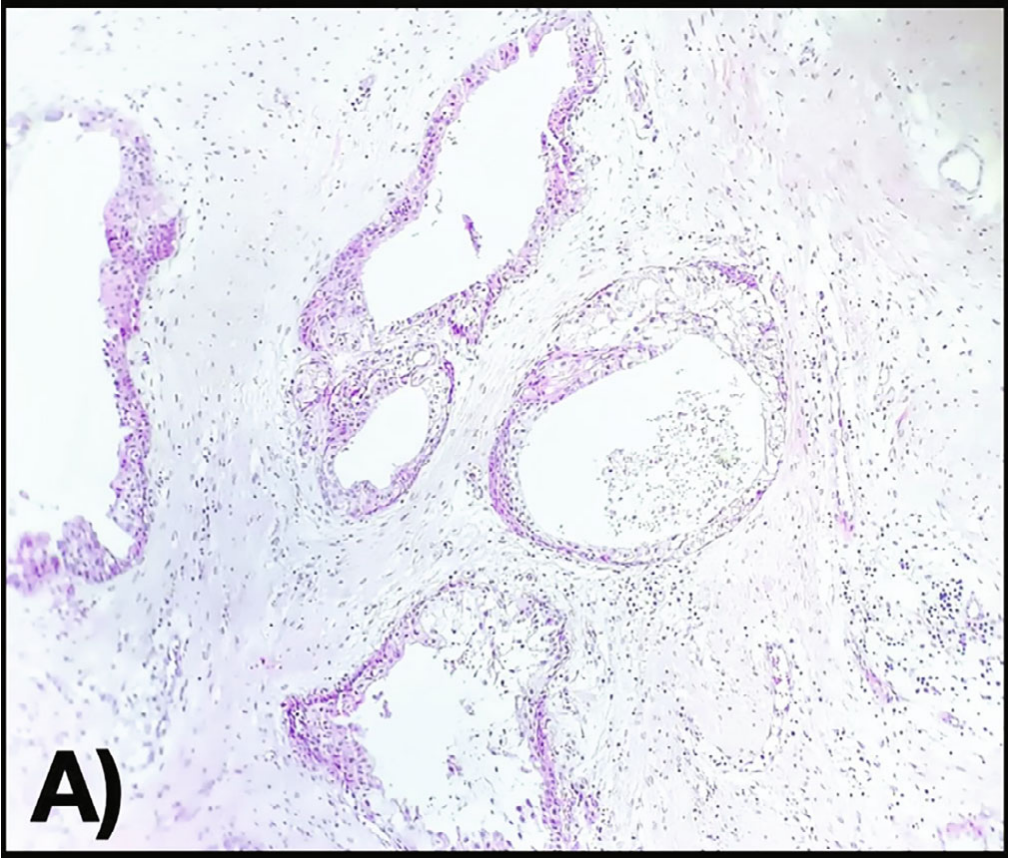

**Figure:**
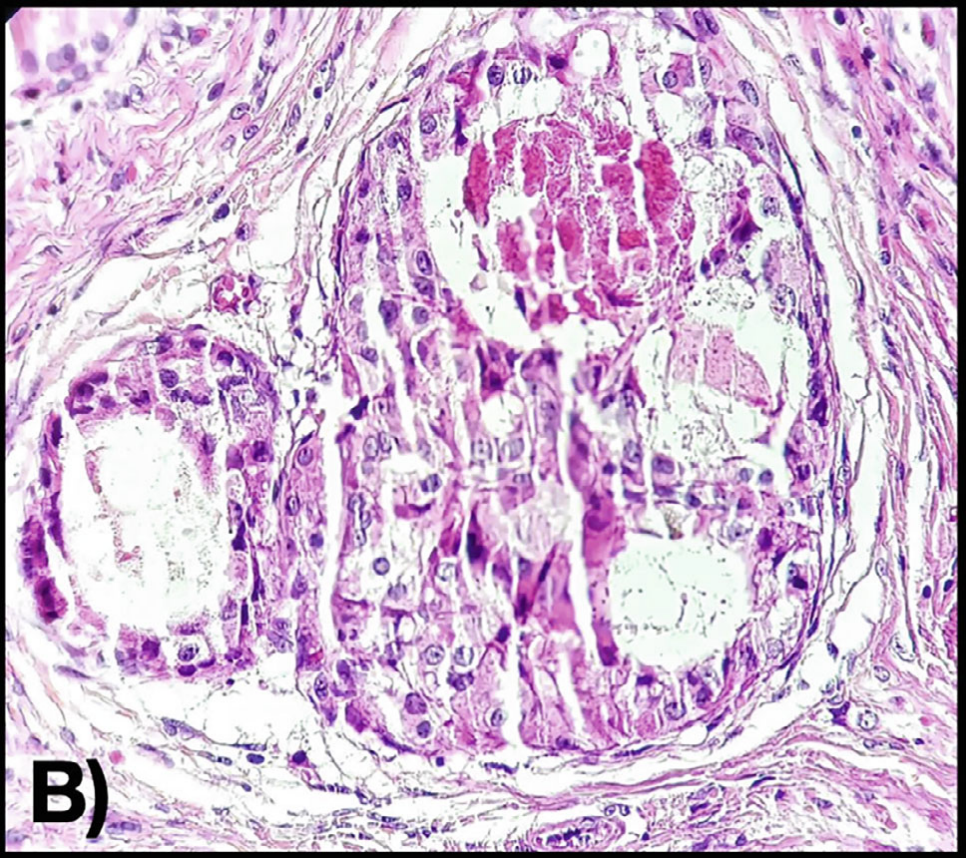

### 3.1. Timeline

The patient underwent operculectomies at ages 16 and 17, with a progressive increase in volume over 1 year leading to biopsy at age 18.

### 3.2. Diagnostic Assessment

Cone beam CT revealed bone destruction adjacent to the mandibular third molar. Histopathology confirmed low‐grade MEC with mucosecretory and epidermoid cells.

### 3.3. Therapeutic Intervention

The patient underwent posterior mandibular block resection with tumor‐free margins.

### 3.4. Follow‐Up and Outcomes

At oncology follow‐up, no recurrence was observed.

### 3.5. Patient Perspective

The patient reported significant fear of dental extractions, leading to a refusal of third molar removal and a preference for operculectomies.

## 4. Discussion

MEC is the most common malignant salivary gland tumor, but it´s occurrence in adolescents is uncommon, representing only about 5% of all cases [1, 8]. The present case highlights an unusual clinical presentation in a young patient, where multiple operculectomies preceded the diagnosis. This raises the question of whether repetitive local trauma or chronic inflammation could act as cofactors in malignant transformation, although current evidence still considers MEC etiology multifactorial and strongly associated with genetic alterations such as the CRTC1‐MAML2 translocation [10, 11]. Compared to typical epidemiological patterns—female prevalence in the fourth to fifth decades and predominant involvement of the parotid gland—our patient deviated from these trends by presenting at 18 years of age with a lesion in the retromolar trigone. This underscores the importance of considering MEC even in atypical demographics and locations, particularly in minor salivary glands, which exhibit higher malignancy potential [7]. Histopathologically, the case was diagnosed as low‐grade MEC, which aligns with the favorable outcome after surgical resection and the absence of recurrence in follow‐up. Recent studies have confirmed that low‐grade and intermediate‐grade MECs have similar survival outcomes, with 5‐year survival rates exceeding 90% as Zhou et al. and Wang et al. reports [9, 12]. However, Sama et al. mentioned high‐grade MEC continues to present significant therapeutic challenges due to higher recurrence and metastasis rates, with survival dropping to approximately 40% [7]. A critical clinical lesson from this case concerns the management of tissue specimens excised during routine dental procedures. Although operculectomy is often performed for pericoronitis, excised tissue is rarely sent for histopathological evaluation. This practice represents a missed diagnostic opportunity, as malignant salivary tumors may mimic benign conditions, such as mucoceles or inflammatory lesions [6]. Therefore, we emphasize the necessity of a standardized protocol mandating histopathological analysis of all oral tissue samples, regardless of size or presumed benign nature. Finally, psychological aspects played a significant role in this case. The patient′s dental anxiety led her to refuse third molar extraction, opting instead for repeated operculectomies, which indirectly delayed diagnosis. In Mexico dental anxiety and phobia are underrecognized barriers to care in adolescents and may contribute to delayed treatment of potentially serious conditions [13]. This highlights the need for integrated approaches in dental care that address both clinical and psychological dimensions, including sedation protocols and patient education. In summary, this case reinforces three key points: (1) MEC should be considered in adolescents with persistent or atypical oral lesions, (2) routine histopathological evaluation of excised oral tissues is essential for early detection, and (3) addressing dental anxiety is crucial to avoid delays in management that may impact prognosis.

## 5. Conclusion

Dental anxiety is a heightened fear of dental procedures that may or may not meet all the criteria for the diagnosis of phobia while phobia is a persistent and excessive fear of dental stimuli and procedures that results in avoidance or significant distress, as occurred with the patient of the presented case. In the most extreme cases, young people with a dental phobia [13] may refuse treatment even when they experience significant pain that could be relieved with appropriate care; however, that does not exempt us from providing the appropriate treatment for the patient.

Pericoronitis/operculitis is defined as the inflammation of the oral soft tissues surrounding the crown of an erupted or partially erupted tooth [14]. Operculectomy is the surgical removal of gum tissue on the partially erupted tooth and is indicated in most dental impactions [1] since they resolve spontaneously when hard and/or soft tissue obstacles are eliminated; however, there are variations in their behavior. Although it´s of minimal incidence, the results can be catastrophic due to poor management.

MEC is a common pathology in adolescents; it´s rare to face malignant tumors in adolescents. However, it´s important to give the same management to the tissues that are removed and send for histopathological study. Due to the emotional history of the patient, it´s important not to detract from the management of anxiety in the dental office, such as having the specialists, techniques, and the necessary equipment to be able to offer the patient intravenous conscious sedation or other management strategies and, above all, protocolize the sending of any tissue sample that is collected. Remove the patient for histopathological study, as set out in the standards with the aim of diagnosing malignant pathologies in the initial stages and being able to offer timely diagnoses with minimally invasive treatments, such as in this case of MEC.

## Conflicts of Interest

The authors declare no conflicts of interest.

## Funding

This study is supported by the Universidad Nacional Autónoma de México (10.13039/501100005739).

## Data Availability

Research data are not shared.
